# Supplementary material for: Estimating time-varying exposure effects through continuous-time modelling in Mendelian randomization
Source: Stat Med. Author manuscript; Available in PMC 2024 Nov 30. (PMC7616825; doi:10.1002/sim.10222)
Supplement: Supplementary file 1 [file EMS199862-supplement-Supplementary_Material.pdf]

Estimating time-varying exposure effects through continuous-time modelling in Mendelian randomization

Supplementary Materials

Haodong Tian[1], Ashish Patel[1], Stephen Burgess[1,2]

[1] MRC Biostatistics Unit, School of Clinical Medicine,
University of Cambridge, Cambridge, UK

[2] British Heart Foundation Cardiovascular Epidemiology Unit,
Department of Public Health and Primary Care,
University of Cambridge, Cambridge, UK

Contents

Supplementary Figures **2**

Supplementary Texts **5**

Text S1: Basic identification equation 5
Text S2: Functional principal component analysis 6
Text S3: Principal components analysis through conditional expectation 7
Text S4: Two-sample summary-level data 8
Text S5: MPCMR estimation with IVW 9
Text S6: Time-varying IV validity and basis function testing 10
Text S7: Time-varying IV strength testing 11
Text S8: Related proof of LM statistic 11
Text S9: Q statistic testing details 12
Text S10: MPCMR with retrospective data and variable time region 14

References **17**

Supplementary Figures

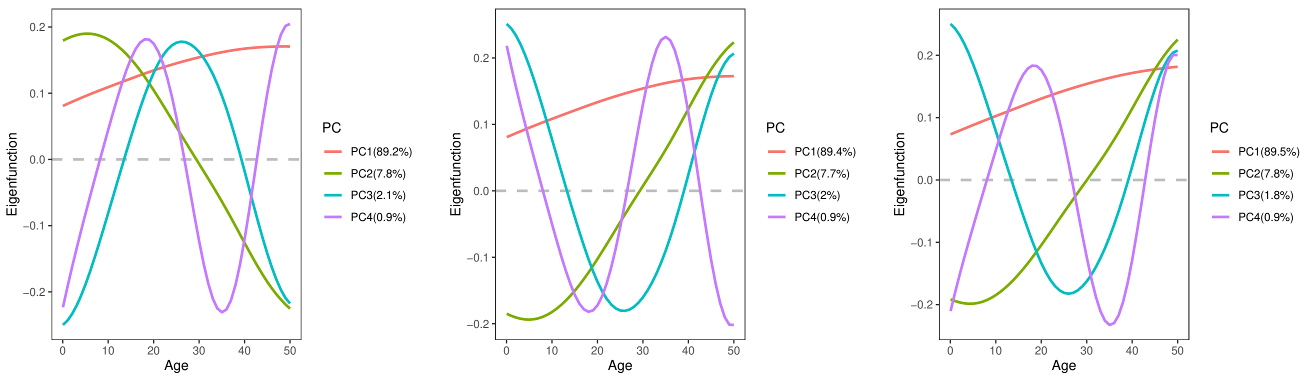

Supplementary Figure A1: The eigenfunction results of functional principal components in one simulation example under the three exposure model scenarios A-C. The left, middle and right panel corresponds to the instrument-exposure scenarios A, B and C, respectively. In all cases, the first and second eigenfunctions, denoted by the red and green curves, are approximately linear and cumulatively explain more than 95% of the variations.

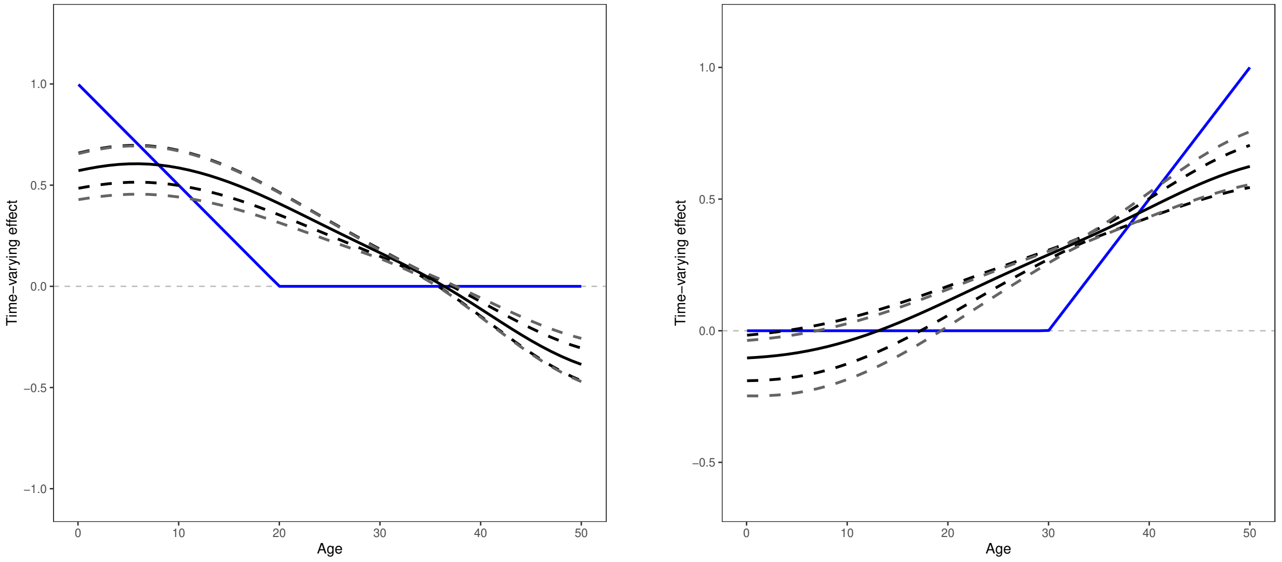

Supplementary Figure A2: The time-varying MR fitting results using eigenfunctions as the basis functions under the exposure model scenario C. Left: the outcome model scenario 5. Right: the outcome model scenario 6. The true effect functions, which are expressed by the blue curves, cannot be represented by the eigenfunctions in an additive linear way, thereby causing bias in most timepoints.

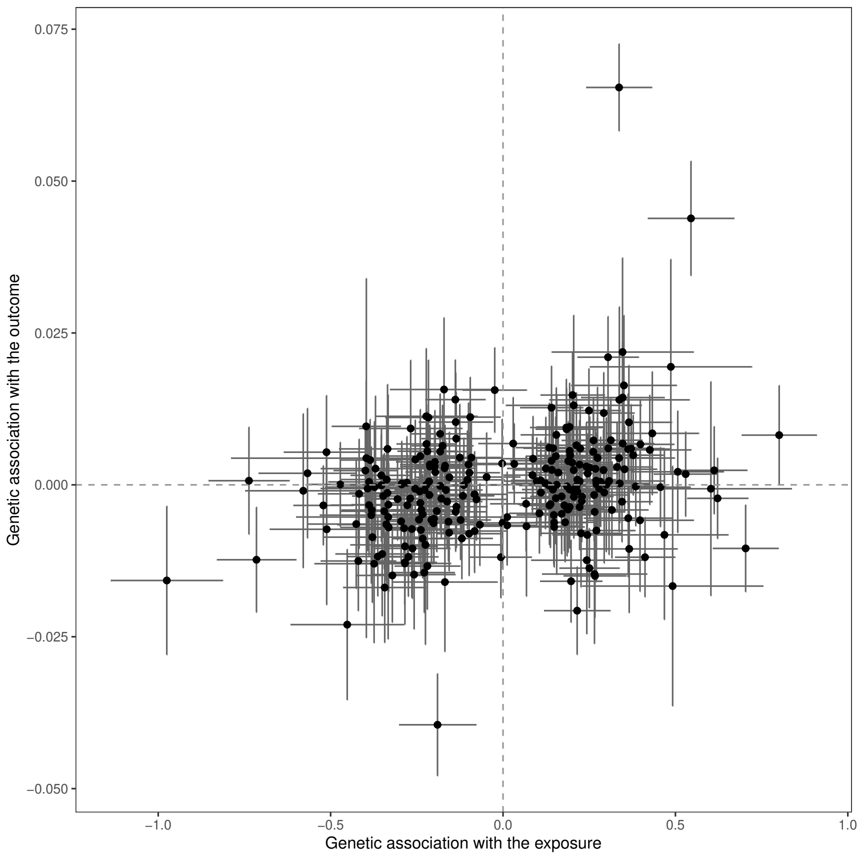

Supplementary Figure A3: Scatter plot of the estimated genetic association with the exposure and outcome for 258 SNPs in the univariable Mendelian randomization fitting.

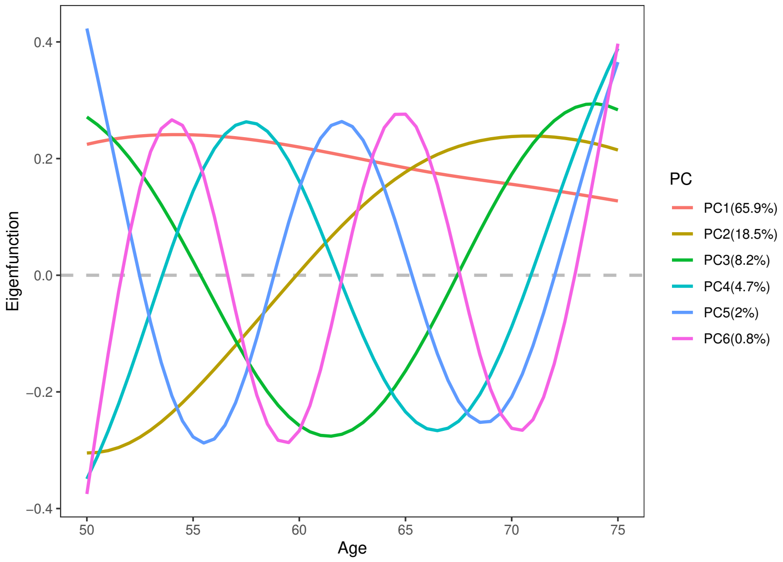

Supplementary Figure A4: The eigenfunctions of FPCA for the SBP data over the age region $[50, 75]$. PC stands for the principal component, followed by the corresponding fraction variance explained.

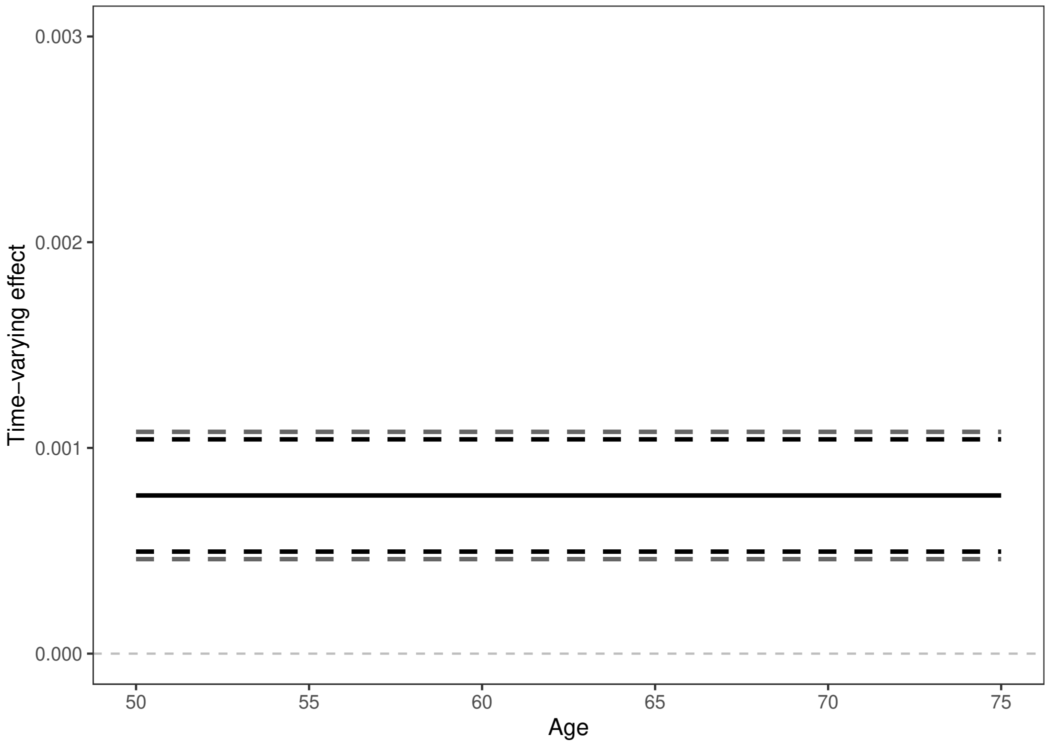

Supplementary Figure A5: Application results using the constant effect. The black curve represents the fitted time-varying effect (here constant effect) from the MPCMR method, while the dashed lines represent 95% confidence intervals derived from GMM (black) and Lagrange multipliers (grey). The effect unit is mmol/L per 1 mmHg higher genetically-predicted SBP.

Supplementary Texts

Text S1: Basic identification equation

We illustrate the key idea of identifying the time-varying effect, which is a functional objective, using instrumental variable (IV) methods. Assuming the IV core assumptions are valid, and the structural outcome is

$$Y_T = \int_0^T \beta_T(t)X(t)dt + g_{Y,T}(U, \epsilon_Y) \tag{S1}$$

which can be regarded as an infinitely dimensional multivariable Mendelian randomization (MVMR) model, where the multiple exposures represent the time-varying exposures at different time points. U contains both the time-invariant and time-varying confounders up to the time point T (e.g. $\{U(t); 0 \leq t \leq T\}$). Let's assume there are J independent genetic variants denoted as $G_j; j = 1, \ldots, J$. The inverse variance weighted (IVW) method involves two-stage fittings. In the second stage, we perform a regression of the outcome on the instruments (with possible covariates for adjustment). The estimated parameter for the j-th instrument follows an asymptotic distribution (here, we ignore the uncertainty associated with the standard errors):

$$\hat{\theta}_j \xrightarrow{D} \mathcal{N}(\theta_j, s.e.(\hat{\theta}_j)^2) \tag{S2}$$

where $\theta_j = cov(Y_T, G_j)/var(G_j) = cov(\int_0^T \beta_T(t)X(t)dt, G_j)/var(G_j) = \int_0^T \beta_T(t)\alpha_j(t)dt$ and $\alpha_j(t) := cov(G_j, X(t))/var(G_j)$. It is easy to know $cov(\hat{\theta}_{j_1}, \hat{\theta}_{j_2}) = 0$ for any $j_1 \neq j_2$. Therefore, we have the following equation that enables the identification of $\beta_T(t)$: $\hat{\theta}_j \sim \mathcal{N}(\int_0^T \beta_T(t)\alpha_j(t)dt, s.e.(\hat{\theta}_j)^2)$, which is the ideal fitting equation for IVW results. However, since $\alpha_j(t)$ is unknown, it needs to be estimated from the data. This estimation can be obtained through the first-stage IVW fitting, which involves regressing $X(t)$ on the instruments (with possible covariates for adjustment). If the uncertainty associated with the estimated functional objective $\hat{\alpha}_j(t)$ is small, we can proceed with the following fitting regression

$$\hat{\theta}_j = \int_0^T \beta_T(t)\hat{\alpha}_j(t)dt + \epsilon_j \qquad \epsilon_j \sim \mathcal{N}(0, s.e.(\hat{\theta}_j)^2) \qquad j = 1, \ldots, J \tag{S3}$$

which is the standard fitting equation for obtaining IVW results. Note that this equation represents an ill-posed inverse problem, which means that $\beta_T(t)$ may not be consistently estimated even if we have a consistent estimator of θ_j and $\alpha_j(t)$ for $j = 1, \ldots, J$. Therefore, we cannot directly express the final estimated term (the effect function, $\hat{\beta}_T(t)$, in time-varying MR) in a manner similar to univariable MR or MVMR without making additional assumptions about $\beta_T(t)$.

Text S2: Functional principal component analysis

We briefly introduce the functional principal component analysis (FPCA) and its basic properties. For more details, see the review paper [21]. Assume the individual function curve $X_i(t), i = 1, \ldots, n$ is a smooth and square-integrable function, where $X_i(t)$ is a functional objective like the exposure trajectory. The FPCA treats $X_i(t)$ as a realization of the random function $X(t)$ over the region of interest $[0, T]$. Denote the mean exposure function by $\mathbb{E}(X(t)) = \mu(t)$. Define the covariance function at any two timepoints (s, t) as $cov(X(s), X(t))$. Assume that the covariance function can be expressed by the orthogonal expansion in the L^2 sense

$$cov(X(s), X(t)) = \sum_{k=1}^{\infty} \lambda_k \phi_k(s) \phi_k(t) \tag{S4}$$

where $\lambda_1 \geqslant \lambda_2 \geqslant \ldots \geqslant \lambda_\infty \geqslant 0$ are called eigenvalues and $\phi_1(\cdot), \phi_2(\cdot), \ldots, \phi_\infty(\cdot)$ are the orthonormal eigenfunctions satisfying in sequence

$$\phi_1 = \underset{\|\phi\|=1}{\arg\max} \left\{ var\left(\int_0^T (X(t) - \mu(t))\phi(t)dt \right) \right\}$$

$$\vdots$$

$$\phi_k = \underset{\|\phi\|=1;<\phi,\phi_j>=0;j=1,\ldots,k-1}{\arg\max} \left\{ var\left(\int_0^T (X(t) - \mu(t))\phi(t)dt \right) \right\}$$

$$\vdots$$

where $\|\phi\| := \left[\int_0^T \phi(t)^2 dt\right]^{\frac{1}{2}}$ and $< \phi, \phi_j >:= \int_0^T \phi(t)\phi_j(t)dt$. The k-th eigenfunction is the function maximizing the variance of the projected value of the exposure curve over this function across individuals and is also orthogonal to the previous eigenfunctions. We define the principal component associated with the k-th eigenfunction as $\xi_k = \int_0^T (X(t) - \mu(t))\phi_k(t)dt$. For any k_1-th and k_2-th principal components, it is easy to know

$$\begin{aligned} cov(\xi_{k_1}, \xi_{k_2}) =& cov(\int_0^T X(t)\phi_{k_1}(t)dt, \int_0^T X(t)\phi_{k_2}(t)dt) = cov(\boldsymbol{\phi}_{k_1}^T \boldsymbol{X}\delta, \boldsymbol{\phi}_{k_2}^T \boldsymbol{X}\delta) = \delta \boldsymbol{\phi}_{k_1}^T cov(\boldsymbol{X}, \boldsymbol{X}) \boldsymbol{\phi}_{k_2}\delta \\ \overset{\langle 1 \rangle}{=}& \delta \boldsymbol{\phi}_{k_1}^T \left(\sum_{k=1}^{\infty} \lambda_k \boldsymbol{\phi}_k \boldsymbol{\phi}_k^T \right) \boldsymbol{\phi}_{k_2}\delta = \sum_{k=1}^{\infty} \lambda_k (\boldsymbol{\phi}_{k_1}^T \boldsymbol{\phi}_k \delta)(\boldsymbol{\phi}_{k_2}^T \boldsymbol{\phi}_k \delta) \\ =& \sum_{k=1}^{\infty} \lambda_k \int_0^T \phi_{k_1}(t)\phi_k(t)dt \int_0^T \phi_{k_2}(t)\phi_k(t)dt = 0 \qquad \text{for } k_1 \neq k_2 \end{aligned} \tag{S5}$$

due to the eigenfunction properties, where $\boldsymbol{\phi}_k = (\phi_k(\delta)\, \phi_k(2\delta) \ldots \phi_k(T))^T$, $\boldsymbol{X} = (X(\delta)\, X(2\delta) \ldots X(T))^T$ and δ is a small time grid for numerical approximation. Equation $\langle 1 \rangle$ is due to the orthogonal expansion (S4).

The variance of principal components in the k-th eigenfunction direction is the k-th eigenvalues. That is,

$var(\xi_k) = \lambda_k$. According to (S4) and the orthonormal eigenfunction property, we can know

$$\int_0^T cov(X(s), X(t))\phi_k(s)ds = \lambda_k\phi_k(t) \tag{S6}$$

The curve can be then expressed by the Karhunen-Loéve theorem as $X(t) - \mu(t) = \sum_{k=1}^{\infty} \xi_k\phi_k(t)$. Hence, we can approximately express each individual curve with the first M eigenfunctions explaining enough variation (e.g., $> 95\%$)

$$X_i(t) \approx \mu(t) + \sum_{m=1}^{M} \xi_{m,i}\phi_m(t) \tag{S7}$$

where $\xi_{m,i}$ is the principal component value of the i-th individual w.r.t the m-th eigenfunction.

Text S3: Principal components analysis through conditional expectation

We briefly introduce how to draw FPCA with sparse measured samples. One can see the original paper [23] proposing PACE in detail. Assume we have the individual data where the individual measured exposure are $\{X_{ij}; j = 1, \ldots, N_i\}$ where X_{ij} represents the j-th observation at timepoint t_{ij} of the i-th individual. PACE makes the following assumptions:

(i) The measured exposure could contain the measurement error for the accurate exposure but it is in an additive independent way; that is, $X_{ij} = X_i(t_{ij}) + \epsilon_{ij}$ and $\epsilon_{ij} \overset{\text{i.i.d}}{\sim} \mathcal{N}(0, \sigma^2)$.

(ii) The individual exposure is a random function expressed as the FPCA (via the Karhunen-Loéve theorem): $X_i(t_{ij}) = \mu(t_{ij}) + \sum_{k=1}^{\infty} \xi_{k,i}\phi_k(t_{ij})$ where the distribution of the principal components $\{\xi_k; k = 1, 2, \ldots\}$ are assumed to be Gaussian.

(iii) The individual measured timepoints $\{t_{ij}; j = 1, \ldots, N_i\}$ and the number of the measured timepoint, N_i, are independent of the measurement errors and the principal components.

Therefore, the individual random variables $\{\boldsymbol{X}_i, \boldsymbol{\xi}_i\}$, where $\boldsymbol{X}_i = (X_{i1} \cdots X_{iN_i})^T$ and $\boldsymbol{\xi}_i = (\xi_{1,i} \cdots \xi_{\infty,i})^T$, follow the multivariate normal distribution:

$$\begin{pmatrix} \boldsymbol{\xi}_i \\ \boldsymbol{X}_i \end{pmatrix} \sim \mathcal{N}(\begin{pmatrix} \mathbf{0} \\ \boldsymbol{\mu}_i \end{pmatrix}, \begin{pmatrix} var(\boldsymbol{\xi}_i) & \boldsymbol{\Gamma} \\ \boldsymbol{\Gamma}^T & \boldsymbol{\Sigma}_{\boldsymbol{X}_i} \end{pmatrix}) \tag{S8}$$

where $\boldsymbol{\Gamma}_{k,j} := cov(\xi_{k,i}, X_i(t_{ij})) = cov(\xi_{k,i}, \xi_{k,i}\phi_k(t_ij)) = \lambda_k\phi_k(t_{ij})$ due to the FPCA property. $\lambda_k = var(\xi_k)$ is the eigenvalue for the k-th principal component, $\boldsymbol{\Sigma}_{\boldsymbol{X}_i} = var(\boldsymbol{X}_i)$, and $\boldsymbol{\mu}_i = (\mu(t_{i1}) \cdots \mu(t_{iN_i}))^T$. The conditional expectation of the k-th principal components given the other observations for an individual is therefore

$$\mathbb{E}(\xi_{k,i}|\boldsymbol{X}_i) = \lambda_k\boldsymbol{\phi}_{k,i}^T\boldsymbol{\Sigma}_{\boldsymbol{X}_i}^{-1}(\boldsymbol{X}_i - \boldsymbol{\mu}_i) \tag{S9}$$

where $\boldsymbol{\phi}_{k,i} = (\phi_k(t_{i1}) \cdots \phi_k(t_{iN_i}))^T$. In practice, we first draw the curve and surface smoothing with observed population data to obtain the estimates $\hat{\mu}(t)$ and $\widehat{cov}(X(t), X(s))$ (as well as $\hat{\sigma}$ and $\hat{\boldsymbol{\Sigma}}_{\boldsymbol{X}}$); then derive the estimated eigenfunctions $\{\hat{\phi}_k(t)\}$ and eigenvalues $\{\hat{\lambda}_k\}$ based on the equation (S6); finally, the conditional expectation of principal components with the plug-in estimates is

$$\hat{\xi}_{k,i} = \hat{\mathbb{E}}(\xi_{k,i}|\boldsymbol{X}_i) = \hat{\lambda}_k \hat{\boldsymbol{\phi}}_{k,i}^T \hat{\boldsymbol{\Sigma}}_{\boldsymbol{X}_i}^{-1}(\boldsymbol{X}_i - \hat{\boldsymbol{\mu}}_i) \tag{S10}$$

which is used as the k-th principal component score of the i-th individual. Similar to before, we can choose the first K principal components for reasonably good approximation and name K as the number of the primary principal components. K can be chosen according to the fraction-of-variance-explained (FVE), cross-validation style, AIC-type criteria, etc.

Text S4: Two-sample summary-level data

Since we transform the time-varying analysis problem into MVMR problems, one advantage of this approach is that it allows for a two-sample and summary-level data setting. This means that the exposure can come from one dataset, while the outcome can come from another dataset [4, 8]. The more flexible data structure significantly increases the applicability of our methods for time-varying studies. In most cases, public longitudinal data do not measure both the exposure and outcome together, as the exposures and outcomes are often defined after the study protocol. In contrast, the most widely recognized models for time-varying studies, namely the structural (nested) mean models [14, 15, 16], typically require one-sample data for fitting. The corresponding IV models [11] also require complete longitudinal information for the instrument, exposure, and outcome.

Our MPCMR models can also be implemented with summary-level data. For summary statistics, we additionally assume that the instrument-exposure model is (where the principal components are either the original PC or the transformed ones with transformation matrix, depending on the choice of basis functions):

$$\xi_k = \alpha_0 + \boldsymbol{\alpha}_k^T \boldsymbol{Z} + V_k \quad k = 1, 2, \ldots, K \tag{S11}$$

with $\mathbb{E}(V_{k1}V_{k2}|\boldsymbol{Z})$ does not depend on $\boldsymbol{Z}$ for any k_1 and k_2, which is called the homoskedasticity assumption. Therefore, we have the instrument-outcome model: $Y = \theta_0 + \boldsymbol{\theta}^T \boldsymbol{Z} + U$, $k = 1, 2, \ldots, K$ with $\mathbb{E}(U|\boldsymbol{Z}) = 0$ and $\mathbb{E}(U^2|\boldsymbol{Z})$ does not depend on $\boldsymbol{Z}$. We can construct the moment functions with summary statistics $\hat{\boldsymbol{g}}(\boldsymbol{\beta}) = \hat{\boldsymbol{\theta}} - \hat{\boldsymbol{\alpha}}\boldsymbol{\beta}$ where the estimated association $\hat{\boldsymbol{\theta}} \in \mathbb{R}^{J\times 1}$ and $\hat{\boldsymbol{\alpha}} \in \mathbb{R}^{J\times K}$ can be from either the univariable regression or multivariable regression. Then the effect function $\beta(t)$ can be estimated with the similar GMM procedures introduced in the main text.

Text S5: MPCMR estimation with IVW

We illustrate the MPCMR estimation with the classical IVW framework. We first assume the effect shape satisfies the parametric form: $\beta(t) = \sum_{l=1}^{L} \gamma_l b_l(t)$ where $\{\beta_l\}$ are known basis functions and $L \le K$ for identification.

Review that the structural equation of MPCMR to form the MVMR respective to the parameters of interest $\{\gamma_l\}$

$$
\begin{aligned}
Y_i =& \beta_0 + \int_0^T \beta(t) X_i(t) dt + g_Y(U_i, \epsilon_{Y,i}) \\
=& \beta_0 + \sum_{l=1}^{L} \gamma_l \int_0^T b_l(t) X_i(t) dt + g_Y(U_i, \epsilon_{Y,i})
\end{aligned}
\tag{S12}
$$

where the MVMR estimates $\hat{\gamma}_l$ is based on the IVW regression, where we assume the SNPs are pruned to be or assumed to be not correlated with each other,

$$
\begin{aligned}
\hat{\theta}_j =& \sum_{l=1}^{L} \gamma_l \frac{cov(G_j, \int_0^T b_l(t) X(t) dt)}{var(G_j)} + \epsilon_j \qquad \epsilon_j \sim \mathcal{N}(0, s.e.(\hat{\theta}_j)^2 \tau^2) \\
=& \sum_{l=1}^{L} \gamma_l \frac{cov(G_j, \int_0^T b_l(t) \left[\mu(t) + \sum_{k=1}^{K} \xi_k \phi_k(t)\right] dt)}{var(G_j)} + \epsilon_j \\
=& \sum_{l=1}^{L} \gamma_l \sum_{k=1}^{K} \frac{cov(G_j, \xi_k)}{var(G_j)} \int_0^T b_l(t) \phi_k(t) dt + \epsilon_j
\end{aligned}
\tag{S13}
$$

with the plug-in estimates $\frac{\widehat{cov}(G_j, \xi_k)}{\widehat{var}(G_j)}$ from the regressing the PCs on the SNPs, and the standard numeric estimates $\int_0^T b_l(t)\phi_k(t) dt \approx \sum_i^I \phi_k(t_i) b_l(t_i) T/I$ for the uniform grids $\{t_i\}$. The IVW regression is therefore

$$
\hat{\boldsymbol{\theta}} = \hat{\boldsymbol{\alpha}} \boldsymbol{B} \boldsymbol{\gamma} + \boldsymbol{\epsilon} \qquad \boldsymbol{\epsilon} \sim \mathcal{N}(\boldsymbol{0}, \tau^2 \boldsymbol{\Sigma}) \tag{S14}
$$

where $\boldsymbol{B} = \int_0^T \boldsymbol{\phi}(t) \boldsymbol{b}^T(t) dt \in \mathbb{R}^{K \times L}$, $\hat{\boldsymbol{\alpha}} \in \mathbb{R}^{J \times K}$ is the instrument-PCs association estimates and $\boldsymbol{\Sigma}$ is the diagonal matrix with $\boldsymbol{\Sigma}_{j,j} = s.e.(\hat{\theta}_j)^2$. The estimators are

$$
\hat{\boldsymbol{\gamma}} = (\boldsymbol{B}^T \hat{\boldsymbol{\alpha}}^T \boldsymbol{\Sigma}^{-1} \hat{\boldsymbol{\alpha}} \boldsymbol{B})^{-1} \boldsymbol{B}^T \hat{\boldsymbol{\alpha}}^T \boldsymbol{\Sigma}^{-1} \hat{\boldsymbol{\theta}} \tag{S15}
$$

with the estimator variance matrix $(\boldsymbol{B}^T \hat{\boldsymbol{\alpha}}^T \boldsymbol{\Sigma}^{-1} \hat{\boldsymbol{\alpha}} \boldsymbol{B})^{-1} \hat{\tau}^2$ and the pointwise functional error $\boldsymbol{b}^T(t) (\boldsymbol{B}^T \hat{\boldsymbol{\alpha}}^T \boldsymbol{\Sigma}^{-1} \hat{\boldsymbol{\alpha}} \boldsymbol{B})^{-1} \hat{\tau}^2 \boldsymbol{b}(t)$. There are typically two common choices for the parametric form of $b_l(t)$:

1. The polynomial basis function: $b_l(t) = t^{l-1}$, $l = 1, 2, \ldots$

2. The eigenfunction system: $b_l = \phi_l(t)$, $l = 1, 2, \ldots$

One special kind of parametric assumption used in recent TVMR studies is to assume the constant-effect form that $\beta(t) = \gamma$ for any timepoint [5], which leads to a one-parameter problem and greatly simplifies the estimation

model. In our context, such an assumption means $\boldsymbol{B} = \int_0^T \boldsymbol{\phi}(t)1dt = \mathbf{1} \in \mathbb{R}^K$ as the eigenfunction property. The IVW regression becomes the UVMR regression model: $\boldsymbol{\theta}_j = \left(\sum_k^K \hat{\alpha}_{j,k}\right)\gamma + \epsilon_j$ where $\epsilon_j \sim \mathcal{N}(0, s.e.(\theta_j)^2)$.

Text S6: Time-varying IV validity and basis function testing

In Mendelian randomization, some genetic variants may be invalid. This can be caused by the possible genetic correlation with confounders (hence the violation of exchangeability), or the genetic pleiotropy phenomenon [7] (hence the violation of the exclusion restriction). One may wish to assess the IV validity. The IV validity test is also called the over-identification test [9], assessing whether all instruments are estimating the same causal effect parameters ($\{\beta_k^*\}$ or $\{\gamma_l\}$). One special source making an instrument invalid is the basis function assumptions for $\beta(t)$. When using the incorrect basis function in $\boldsymbol{B}$, the instrument for the transformed principal components will fail to estimate the same parameter and therefore be invalid. When some instrument is invalid, the over-identification should be rejected. Note that we cannot test the validity when all instruments used are invalid in the same way, therefore caution should be given that the IV validity test assesses only the coherency rather than validity [12]. However, due to the ignorable possibility of all instruments being invalid in the same way, we can regard the over-identification test as the IV validity test.

We can test the IV validity using many methods, including Cochran's Q test [6], Sargan J test [20], or other goodness-of-fit tests. We take the Q test as an example due to its popular usage for summary-level MR analysis. Assume the null hypothesis H_0: all instruments are valid and $\beta(t) = \sum_{l=1}^L \gamma_l b_l(t)$ is the correct shape form. We assume that each single instrument is uncorrelated with each other. This independent instrument assumption can be guaranteed in practice by pruning the SNPs to near independence (e.g. by using the genetics analysis software PLINK [13]) or compressing them to principal components by ordinary PCA [2]. When independent instruments cannot be guaranteed, one can choose the Sargan J test instead (this is also embedded in our package TVMR). Let the number of instruments and the (untransformed) PCs be J and K (for identification, we require $J \geq K \geq L$). The corresponding test statistic is:

$$Q = \sum_{j=1}^{J} \frac{(\hat{\theta}_j - \hat{\boldsymbol{\alpha}}_j^T \boldsymbol{B}\hat{\boldsymbol{\gamma}})^2}{s.e.(\hat{\theta}_j)^2 + \hat{\boldsymbol{\gamma}}^T \boldsymbol{B}^T \boldsymbol{\Sigma}_{\alpha,j} \boldsymbol{B}\hat{\boldsymbol{\gamma}} - 2\hat{\boldsymbol{\gamma}}^T \boldsymbol{B}^T \boldsymbol{\Gamma}_j} \tag{S16}$$

where $\hat{\boldsymbol{\alpha}}_j$ is the estimated instrument-PCs associations for the j-th instrument, and $\boldsymbol{\Sigma}_{\alpha,j}$ is the corresponding variance matrix. $\boldsymbol{\Gamma}_j = \text{cov}(\hat{\boldsymbol{\alpha}}_j, \hat{\theta}_j)$. Under the null hypothesis H_0 that the instruments are valid and the basis function assumption is correctly specified, the statistic follows a χ^2_{J-L} distribution asymptotically. The details for calculating each element in this statistic can be seen in Supplementary Text S9. Particularly, the quantities $\hat{\gamma}$ can be estimated by either the maximal profile likelihood procedure [24, 22] or the iterative steps (given in Supplementary Text S9) that are commonly used in summary-data MR studies [3].

Note that one can use the statistic (S16) to test the IV validity only, regardless of the basis function assumptions.

This is because, no matter the form of $\beta(t)$, the instruments should estimate the same parameters $\{\beta_k^*\}$ under the null hypothesis. This is equivalent to the classical MVMR model, in which case the Q statistic equals the formula (S16) by setting $\boldsymbol{B} = \boldsymbol{I}$ or any other transformation matrix $\boldsymbol{B} \in \mathbb{R}^{K \times K}$ that is full rank.

Text S7: Time-varying IV strength testing

Another critical problem in Mendelian randomization and instrumental variable analysis is the weak instrument. The weak instrument refers to the instrument's strength being insufficient to support meaningful and reliable results. A weak instrument can lead to ill-identification problems and make the inference on the parameters of interest invalid and unreliable [1]. When fitting univariable MR models, the strength of the instrument depends on the strength and uncertainty of the genetic associations, which can be expressed by its effect size or the F statistics [10, ch. 16]. When fitting multivariable MR models, the concept of a weak instrument needs to take into account the possible linear dependence that the genetic association with a single exposure (PC in our context) may be linearly expressed by the genetic association with the remaining exposures (PCs). This leads to larger estimation error and, therefore, weak instrument bias. Such linear dependence can be expressed by either conditional F statistics [19, 17] or the Q statistic [18]. The same test statistic (S16) can be used for testing the instrument strength.

When using the Q statistic to test the instrument strength for the k-th PC with the remaining PCs, we treat the k-th PC as the 'outcome,' and the remaining PCs are the 'exposures.' We then let $\hat{\theta}_j = \hat{\boldsymbol{\alpha}}_{j,k}$ and use the updated $\hat{\boldsymbol{\alpha}}_j \leftarrow \hat{\boldsymbol{\alpha}}_{j,-k}$ with the corresponding adjusted standard errors. Set $\boldsymbol{B} = \boldsymbol{I} \in \mathbb{R}^{(K-1)\times(K-1)}$, and the testing degree of freedom is $J - (K-1)$. Strong evidence for rejection indicates strong instrument strength.

The IV strength and validity test is provided by our package TVMR.

Text S8: Related proof of LM statistic

We show that $\hat{\Delta}_k = \hat{\mathbb{E}}_n(-\boldsymbol{Z}\xi_k(Y - \boldsymbol{\xi}^T\boldsymbol{\beta}_0)\boldsymbol{Z}^T)$ is the consistent estimator of $\Delta_k := cov(\sqrt{n}\hat{\boldsymbol{g}}(\boldsymbol{\beta}_0), \sqrt{n}\hat{\boldsymbol{G}}_k)$.

Proof. Without loss of generality, we let all variables be centered; hence

$$\begin{aligned}\Delta_k =& cov(\sqrt{n}\hat{\boldsymbol{g}}(\boldsymbol{\beta}_0), \sqrt{n}\hat{\boldsymbol{G}}_k) \\ =& n\, cov(\hat{\mathbb{E}}_n(\boldsymbol{Z}(Y - \boldsymbol{\xi}^T\boldsymbol{\beta}_0)), \hat{\mathbb{E}}_n(-\boldsymbol{Z}\xi_k)) \\ =& cov(\boldsymbol{Z}(Y - \boldsymbol{\xi}^T\boldsymbol{\beta}_0), -\boldsymbol{Z}\xi_k) \quad \text{due to i.i.d samples} \\ =& \mathbb{E}(-\boldsymbol{Z}\xi_k(Y - \boldsymbol{\xi}^T\boldsymbol{\beta}_0)\boldsymbol{Z}^T)\end{aligned} \tag{S17}$$

□

We show that $cov(\sqrt{n}\hat{D}_k(\boldsymbol{\beta}_0), \sqrt{n}\hat{\boldsymbol{g}}(\boldsymbol{\beta}_0)) \xrightarrow{p} \boldsymbol{0}$.

Proof. review that $\hat{D}_k(\boldsymbol{\beta}_0) = \hat{\boldsymbol{G}}_k - \hat{\Delta}_k(\boldsymbol{\beta}_0)^T\hat{\Omega}(\boldsymbol{\beta}_0)^{-1}\hat{\boldsymbol{g}}(\boldsymbol{\beta}_0)$; hence

$$\begin{aligned} cov(\sqrt{n}\hat{D}_k(\boldsymbol{\beta}_0), \sqrt{n}\hat{\boldsymbol{g}}(\boldsymbol{\beta}_0)) =& cov(\sqrt{n}\hat{\boldsymbol{G}}_k, \sqrt{n}\hat{\boldsymbol{g}}(\boldsymbol{\beta}_0)) - cov(\sqrt{n}\hat{\Delta}_k(\boldsymbol{\beta}_0)^T\hat{\Omega}(\boldsymbol{\beta}_0)^{-1}\hat{\boldsymbol{g}}(\boldsymbol{\beta}_0), \sqrt{n}\hat{\boldsymbol{g}}(\boldsymbol{\beta}_0)) \\ \xrightarrow{p}& cov(\sqrt{n}\hat{\boldsymbol{G}}_k, \sqrt{n}\hat{\boldsymbol{g}}(\boldsymbol{\beta}_0)) - \Delta_k(\boldsymbol{\beta}_0)^T\Omega(\boldsymbol{\beta}_0)^{-1}cov(\sqrt{n}\hat{\boldsymbol{g}}(\boldsymbol{\beta}_0), \sqrt{n}\hat{\boldsymbol{g}}(\boldsymbol{\beta}_0)) \\ =& \Delta_k(\boldsymbol{\beta}_0)^T - \Delta_k(\boldsymbol{\beta}_0)^T\Omega(\boldsymbol{\beta}_0)^{-1}\Omega(\boldsymbol{\beta}_0) \quad \text{by definition} \\ =& \mathbf{0} \end{aligned} \tag{S18}$$

□

Text S9: Q statistic testing details

We provide the testing inference details regarding the parametric MPCMR models. For UVMR, a similar inference procedure can be found in the papers [24, 3]; for MVMR, see the papers [22, 18]. Assume the null hypothesis H_0 is: $\beta(t) = \sum_{l=1}^{L} \gamma_l b_l(t)$ for fixed basis functions $\{b_l(t)\}$ and parameters $\{\gamma\}$ to be estimated. We assume that the SNPs are pruned to be uncorrelated to each other; therefore, the covariance of the genetic associations from different SNPs to any phenotypes (PCs, outcome, whether same or different) is neglectably zero. We can build the joint likelihood for the estimated associations with the phenotypes for each SNP

$$\begin{pmatrix} \hat{\theta}_j \\ \hat{\boldsymbol{\alpha}}_j \end{pmatrix} \sim \mathcal{N}(\begin{pmatrix} \boldsymbol{\alpha}_j^T\boldsymbol{B}\boldsymbol{\gamma} \\ \boldsymbol{\alpha}_j \end{pmatrix}, \begin{pmatrix} s.e.(\hat{\theta}_j)^2 & \boldsymbol{\Gamma}_j^T \\ \boldsymbol{\Gamma}_j & \boldsymbol{\Sigma}_{\alpha,j} \end{pmatrix}) \qquad j = 1, 2, \ldots, J \tag{S19}$$

Where $\boldsymbol{\Sigma}_{\alpha,j}$ is the variance matrix for $\hat{\boldsymbol{\alpha}}_j$. We can approximate that $\boldsymbol{\Sigma}_{\alpha,j}$ is a diagonal matrix with the k-th element equal to $s.e.(\hat{\alpha}_{j,k})^2$. $\boldsymbol{\Gamma}_j := cov(\hat{\boldsymbol{\alpha}}_j, \hat{\theta}_j)$. When the exposure and the outcome come from two samples, $\boldsymbol{\Gamma}_j = \mathbf{0}$; otherwise $\boldsymbol{\Gamma}_j$ is estimated by the approximation under the overlapping-samples setting

$$\boldsymbol{\Gamma}_{j,k} = cov(\hat{\alpha}_{j,k}, \hat{\theta}_j) = \frac{n_s}{n_1 n_2}\frac{1}{var(G_j)}cov(v_k^{PC}, v^Y) \tag{S20}$$

with the relevant plug-in estimates, where v_k^{PC} and v^Y are the residuals of the regression on the genetic variants from the k-th PC and the outcome, respectively, under the common dataset; n_1 and n_2 are the sample size of the PC data and the outcome data, n_s is the shared sample size.

Proof. Assume the genetic variants are uncorrelated with each other. Consider the regression for the two phenotypes ξ_k and Y with the respective (possibly overlapping) samples:

$$\xi_{k,i} = \alpha_0 + \boldsymbol{\alpha}^T\boldsymbol{G}_i + v_{1,i} \qquad i = 1, \ldots, n_1 \tag{S21}$$

$$Y_i = \theta_0 + \boldsymbol{\theta}^T\boldsymbol{G}_i + v_{2,i} \qquad i = 1, \ldots, n_2 \tag{S22}$$

where $v_1 \overset{\text{def}}{=} v_k^{PC}$ and $v_2 \overset{\text{def}}{=} v^Y$; $\alpha_j = cov(G_j, \xi_k)/var(G_j)$ and $\theta_j = cov(G_j, Y)/var(G_j)$. When $\boldsymbol{G}$ are binary (like in most MR cases), it is easy to know $\mathbb{E}(G^p v_1) = 0$ and $\mathbb{E}(G^p v_2) = 0$ for any order $p = 0, 1, \ldots$. Therefore, the genetic association estimators for the j-th genetic variant are

$$\hat{\alpha}_{j,k} = \frac{\widehat{cov}(G_j, \xi_k)}{\widehat{var}(G_k)} = \frac{(\overline{G_j \xi_k})_{n_1} - (\overline{G_j})_{n_1}(\overline{\xi_k})_{n_1}}{\widehat{var}(G_k)} \tag{S23}$$

$$\hat{\theta}_j = \frac{\widehat{cov}(G_j, Y)}{\widehat{var}(G_k)} = \frac{(\overline{G_j Y})_{n_2} - (\overline{G_j})_{n_2}(\overline{Y})_{n_2}}{\widehat{var}(G_k)} \tag{S24}$$

The approximation has the following steps

$$\begin{aligned}
cov(\hat{\alpha}_{j,k}, \hat{\theta}_j) &\overset{\langle 1 \rangle}{=} \frac{1}{var(G_j)^2} cov[(\overline{G_j \xi_k})_{n_1} - (\overline{G_j})_{n_1}(\overline{\xi_k})_{n_1}, (\overline{G_j Y})_{n_1} - (\overline{G_j})_{n_1}(\overline{Y})_{n_1}] \\
&= \frac{1}{var(G_j)^2} cov[\frac{1}{n_1}\sum_{i=1}^{n_1} G_{j,i}(\xi_{k,i}^C + (\overline{\xi_k})_{n_1}) - (\overline{G_j})_{n_1}(\overline{\xi_k})_{n_1}, \frac{1}{n_2}\sum_{i=1}^{n_1} G_{j,i}(Y_i^C + (\overline{Y})_{n_2}) - (\overline{G_j})_{n_2}(\overline{Y})_{n_2}] \\
&\overset{\langle 2 \rangle}{=} \frac{1}{var(G_j)^2} cov[\frac{1}{n_1}\sum_{i=1}^{n_1} G_{j,i}\xi_{k,i}^C, \frac{1}{n_2}\sum_{i=1}^{n_2} G_{j,i} Y_i^C] \\
&\overset{\langle 3 \rangle}{=} \frac{n_s}{n_1 n_2} \frac{1}{var(G_j)^2} cov(G_j \xi_k^C, G_j Y^C) \\
&\overset{\langle 4 \rangle}{=} \frac{n_s}{n_1 n_2} \frac{1}{var(G_j)^2} cov(\alpha_{j,k} G_j^2 + G_j v_1, \theta_j G_j^2 + G_j v_2) \\
&= \frac{n_s}{n_1 n_2} \frac{1}{var(G_j)^2} \left(\alpha_{j,k}\theta_j var(G_j^2) + cov(G_j v_1, G_j v_2)\right) \\
&\overset{\langle 5 \rangle}{\approx} \frac{n_s}{n_1 n_2} \frac{1}{var(G_j)^2} cov(G_j v_1, G_j v_2) \\
&\overset{\langle 6 \rangle}{=} \frac{n_s}{n_1 n_2} \frac{1}{var(G_j)} cov(v_1, v_2)
\end{aligned} \tag{S25}$$

Where the centred phenotypes ξ_k^C and Y^C in Equation $\langle 2 \rangle$ corresponds to the equations $\xi_k - \mathbb{E}(\xi_k) = \boldsymbol{\alpha}^T \boldsymbol{G} + v_1$ and $Y - \mathbb{E}(Y) = \boldsymbol{\theta}^T \boldsymbol{G} + v_2$, and n_s in Equation $\langle 3 \rangle$ is the shared sample size. In Equation $\langle 4 \rangle$, we assume $\mathbb{E}(G_j^2 G_{j_*} v_1) = 0$ and $\mathbb{E}(G_j^2 G_{j_*} v_2) = 0$ for any j_*. We approximate $\alpha_{j,k}\theta_j \approx 0$ in Equation $\langle 5 \rangle$, as the genetic associations in Mendelian randomization are usually small. In equation $\langle 6 \rangle$, we assume $\mathbb{E}(G_j^2 v_1 v_2) = \mathbb{E}(G_j^2)\mathbb{E}(v_1 v_2)$, which equals to $var(G_j)cov(v_1, v_2)$. □

Hence, the estimator for $\boldsymbol{\Gamma}_{j,k}$ is

$$\hat{\boldsymbol{\Gamma}}_{j,k} = \widehat{cov}(\hat{\alpha}_{j,k}, \hat{\theta}_j) = \frac{n_s}{n_1 n_2} \frac{1}{\widehat{var}(G_j)} \widehat{cov}(v_k^{PC}, v^Y) \approx \frac{1}{n_1 n_2} \frac{1}{\widehat{var}(G_j)} \sum_{i=1}^{n_s} (\xi_{k,i} - \hat{\xi}_{k,i})(Y_i - \hat{Y}_i) \qquad k = 1, \ldots, K \tag{S26}$$

where $\widehat{cov}(v_k^{PC}, v^Y)$ is estimated by the share sample part, and we can approximate that $\frac{1}{n_s}\sum_{i=1}^{n_s}(\xi_{k,i} - \hat{\xi}_{k,i}) \sum_{i=1}^{n_s}(Y_i -$

$\hat{Y}_i) \approx 0$. The statistic is therefore

$$Q = \sum_{j=1}^{J} \frac{(\hat{\theta}_j - \hat{\boldsymbol{\alpha}}_j^T \boldsymbol{B}\hat{\boldsymbol{\gamma}})^2}{s.e.(\hat{\theta}_j)^2 + \hat{\boldsymbol{\gamma}}^T \boldsymbol{B}^T \boldsymbol{\Sigma}_{\alpha,j} \boldsymbol{B}\hat{\boldsymbol{\gamma}} - 2\hat{\boldsymbol{\gamma}}^T \boldsymbol{B}^T \boldsymbol{\Gamma}_j} \tag{S27}$$

Under the null that the parametric model is correctly specified, the statistic follows the chi-squared distribution χ^2_{J-L}. The estimator of $\hat{\boldsymbol{\gamma}}$ based on the statistic is regarded as the robust estimator. The iterative steps for deriving the robust estimator and Q statistic is

(1) Obtain the initial estimates $\hat{\boldsymbol{\gamma}}_{(0)}$ based on Equation (S15), which is to minimize

$$Q(\boldsymbol{\gamma}; \boldsymbol{\gamma}_*) = \sum_{j=1}^{J} \frac{(\hat{\theta}_j - \hat{\boldsymbol{\alpha}}_j^T \boldsymbol{B}\boldsymbol{\gamma})^2}{s.e.(\hat{\theta}_j)^2 + \boldsymbol{\gamma}_*^T \boldsymbol{B}^T \boldsymbol{\Sigma}_{\alpha,j} \boldsymbol{B}\boldsymbol{\gamma}_* - 2\boldsymbol{\gamma}_*^T \boldsymbol{B}^T \boldsymbol{\Gamma}} \tag{S28}$$

with $\boldsymbol{\gamma}_* = \mathbf{0}$ (this is equivalent to the standard MVMR fitting).

(2) Minimize $Q(\boldsymbol{\gamma}; \hat{\boldsymbol{\gamma}}_{(0)})$ to obtain $\hat{\boldsymbol{\gamma}}_{(1)}$

(3) Repeat the above step and update the iterative estimates $\hat{\boldsymbol{\gamma}}_{(n)}$ until the estimates are stable.

The corresponding Q statistic value with the plug-in MPCMR robust estimates is then used to evaluate the parametric shape assumptions or other testing objectives (like IV strength and validity as introduced in the previous supplementary texts).

Text S10: MPCMR with retrospective data and variable time region

In the main text, we propose the framework using the same (fixed) time region, $[0, T]$, for all individuals and conducted FPCA over the time region. The time 0 and time T are defined as the minimal and maximal time points supported by the available data so that FPCA can be well-conducted. However, many longitudinal cohort studies have a much wider range of baseline ages and varying follow-up times due to censoring. This could make FPCA harder to conduct over a fixed time region due to the more limited data over the fixed time region.

In this section, we provide the illustration of conducting MPCMR analysis with other data types and variable time regions. The key feature is that FPCA can be conducted over a larger time region using more data information but the time region will be defined differently for different individuals (i.e. variable time region).

One possible data scenario is that the exposure and outcome are recorded at the current time point (e.g. age) and the exposure trajectory is recorded retrospectively in multiple past time points for each individual (i.e. retrospective data). In this scenario, the current time point can be different among individuals and we may not be able to find a fixed large time region $[0, T]$ as the main text such that FPCA can be well conducted. However, it is still possible to conduct MPCMR using the variable time regions taking the individual current time information into account. Here we give an introduction to the model and estimation with retrospective data.

The structural equation model for the outcome at the individual current age, T_i, is

$$Y_i := Y_i(T_i) = \beta_0(T_i) + \int_0^L \beta_{T_i}(T_i - L + t)X_i(T_i - L + t)dt + U_i(T_i) \tag{S29}$$

where the subscript T_i for $\beta_{T_i}(\cdot)$ reminds us that the time-varying effect is defined with respect to the outcome at the time point T_i. We assume there is no direct effect of past exposure that is more than L ($L < T_i$ for any i) time long from the present time on the current outcome. We are interested to the time-varying effect $\beta(\cdot)$, either the outcome-age-specific function $\beta_T(T - L + t)$ with ang given T or its mean function $\mathbb{E}_T(\beta_T(T - L + t))$, which is defined as $\beta^\dagger(t)$. Note that if the current age T_i is similar to each individual of this data, we can ignore the outcome age difference and the model (S29) is then simplified to the structural outcome equation introduced by the main text (in the main text, we use T representing L instead).

Let the new time-varying exposure be $X^*(t) := X(T - L + t)$ defined over $t \in [0, T]$. FPCA can find the approximation for each individual $X_i^*(t) := X_i(T_i - L + t) = \mu(t) + \sum_{k=1}^K \xi_{k,i}\phi_k(t) + v_i(t)$ for a K with sufficient variance explained and small $v_i(t)$. The FPCA for MPCMR with such a scenario is demonstrated by Figure A6.

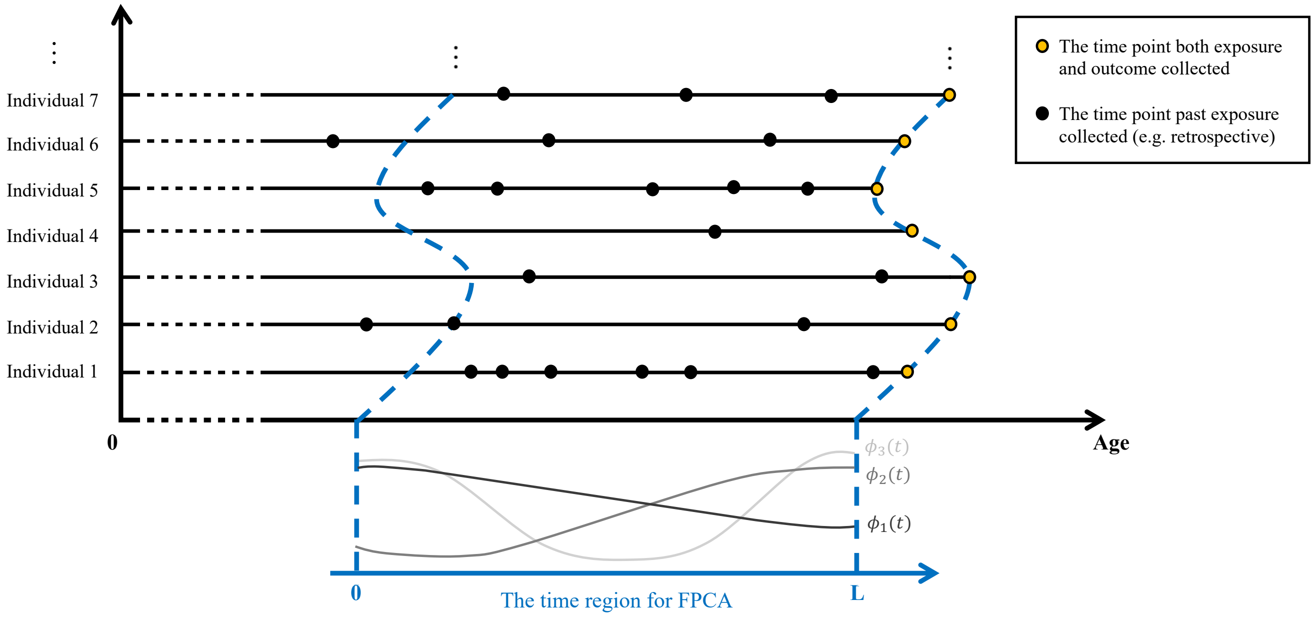

Supplementary Figure A6: The demonstration of the study design and FPCA for the data scenario where each individual can have a different time region with different end time points. The outcome level is collected for all individuals at their current time point (or age). A time length L was used for building the retrospective time region over which the past exposure is collected (indicated by the parallel dashed blue curves). This time region is then used for FPCA to obtain the eigenfunctions (the grey curves $\{\phi_1(t), \phi_2(t), \phi_3(t)\}$) as well as the individual principal components. In this retrospective time scenario, the outcome is precisely measured at the end time point so unlike the real example of the main text approximation for the outcome is not needed. FPCA: functional principal component analysis.

Following the similar estimation strategies introduced by the main text, we have

$$
\begin{aligned}
Y_i =&\beta_0(T_i) + \int_0^L \beta_{T_i}(T_i - L + t)X_i(T_i - L + t)dt + U_i(T_i) \\
=&\beta_0(T_i) + \int_0^L \beta_{T_i}(T_i - L + t)\left[\mu(t) + \sum_{k=1}^K \xi_{k,i}\phi_k(t) + v_i(t)\right] dt + U_i(T_i) \qquad \text{via FPCA} \\
=&\underbrace{\beta_0(T_i) + \int_0^L \beta_{T_i}(T_i - L + t)\mu(t)dt}_{=:\beta_0^*(T_i)} + \int_0^L \beta_{T_i}(T_i - L + t)\left[\sum_{k=1}^K \xi_{k,i}\phi_k(t)\right] dt + \underbrace{\int_0^T \beta_{T_i}(T_i - L + t)v_i(t)dt + U_i(T_i)}_{\approx U_i(T_i)} \\
=&\beta_0^*(T_i) + \sum_{k=1}^K \underbrace{\left[\int_0^L \beta_{T_i}(T_i - L + t)\phi_k(t)dt\right]}_{=:\beta_k^*(T_i)} \xi_{k,i} + U_i(T_i)
\end{aligned}
\tag{S30}
$$

This means that the model can be regarded as an MVMR model with the pseudo-exposures, $\{\xi_k\}$, and their age-modified 'effect' on the outcome, $\{\beta_k^*(T_i)\}$ (here the age T_i can be regarded as a covariate). Given the condition that the instrument is independent of T, any IV estimation methods with valid instruments can therefore identify the average 'effect' of the pseudo-exposures; that is,

$$
\mathbb{E}[\beta_k^*(T)] = \mathbb{E}[\int_0^L \beta_{T_i}(T_i - L + t)\phi_k(t)dt] = \int_0^L \mathbb{E}[\beta_{T_i}(T_i - L + t)]\phi_k(t)dt = \int_0^L \beta^\dagger(t)\phi_k(t)dt \tag{S31}
$$

Therefore, when the objective is the average effect function $\beta^\dagger(t)$, all the estimating and inference procedures are essentially the same as the procedure illustrated in the main text, and the only difference is the final interpretation of the effect function, where its value at the time point t represents the average effect of the exposure at the $(L-t)$ time long earlier than the current outcome age on the outcome over the distribution of age in the population. In practice, one may assume the average effect function $\beta^\dagger(t)$ as the linear additive function where the basis functions are the first two eigenfunctions (similar to those in the simulation and real application of the main text). Alternatively, when one wishes to have more insights on the detailed time-varying effect of age information, one may assume a parametric model taking into account the age information T for the time-varying effect function $\beta_T(\cdot)$; for example, $\beta_{T_i}(T_i - L + t) = \gamma_0 + \gamma_1 T_i + \gamma_2 t$, which is similar to the MPCMR with generalized transformed exposures illustrated in the main text for the parameters $\{\gamma_0, \gamma_1, \gamma_2\}$ where we now have the additional transformed exposures due to the age information, $\sum_{k=1}^K T_i[\int_0^T \phi_k(t)dt]\xi_{k,i}$.

Appendix References

[1] Isaiah Andrews, James H Stock, and Liyang Sun. Weak instruments in instrumental variables regression: Theory and practice. *Annual Review of Economics*, 11:727–753, 2019.

[2] Fatima Batool, Ashish Patel, Dipender Gill, and Stephen Burgess. Disentangling the effects of traits with shared clustered genetic predictors using multivariable mendelian randomization. *Genetic Epidemiology*, 46(7):415–429, 2022.

[3] Jack Bowden, Fabiola Del Greco M, Cosetta Minelli, Qingyuan Zhao, Debbie A Lawlor, Nuala A Sheehan, John Thompson, and George Davey Smith. Improving the accuracy of two-sample summary-data mendelian randomization: moving beyond the nome assumption. *International journal of epidemiology*, 48(3):728–742, 2019.

[4] Stephen Burgess, Robert A Scott, Nicholas J Timpson, George Davey Smith, Simon G Thompson, and EPIC-InterAct Consortium. Using published data in mendelian randomization: a blueprint for efficient identification of causal risk factors. *European journal of epidemiology*, 30:543–552, 2015.

[5] Ying Cao, Suja S Rajan, and Peng Wei. Mendelian randomization analysis of a time-varying exposure for binary disease outcomes using functional data analysis methods. *Genetic epidemiology*, 40(8):744–755, 2016.

[6] William G Cochran. The combination of estimates from different experiments. *Biometrics*, 10(1):101–129, 1954.

[7] George Davey Smith and Shah Ebrahim. 'mendelian randomization': can genetic epidemiology contribute to understanding environmental determinants of disease? *International journal of epidemiology*, 32(1):1–22, 2003.

[8] Neil M Davies, Michael V Holmes, and George Davey Smith. Reading mendelian randomisation studies: a guide, glossary, and checklist for clinicians. *bmj*, 362, 2018.

[9] Lars Peter Hansen. Large sample properties of generalized method of moments estimators. *Econometrica: Journal of the econometric society*, pages 1029–1054, 1982.

[10] MA Hernan and J Robins. Causal inference: What if. boca raton: Chapman & hill/crc. 2020.

[11] Haben Michael, Yifan Cui, Scott A Lorch, and Eric J Tchetgen Tchetgen. Instrumental variable estimation of marginal structural mean models for time-varying treatment. *Journal of the American Statistical Association*, pages 1–12, 2023.

[12] Paulo MDC Parente and JMC Santos Silva. A cautionary note on tests of overidentifying restrictions. *Economics Letters*, 115(2):314–317, 2012.

[13] Shaun Purcell, Benjamin Neale, Kathe Todd-Brown, Lori Thomas, Manuel AR Ferreira, David Bender, Julian Maller, Pamela Sklar, Paul IW De Bakker, Mark J Daly, et al. Plink: a tool set for whole-genome association and population-based linkage analyses. *The American journal of human genetics*, 81(3):559–575, 2007.

[14] James M Robins. The analysis of randomized and non-randomized aids treatment trials using a new approach to causal inference in longitudinal studies. *Health service research methodology: a focus on AIDS*, pages 113–159, 1989.

[15] James M Robins. Correcting for non-compliance in randomized trials using structural nested mean models. *Communications in Statistics-Theory and methods*, 23(8):2379–2412, 1994.

[16] James M Robins. Marginal structural models versus structural nested models as tools for causal inference. In *Statistical models in epidemiology, the environment, and clinical trials*, pages 95–133. Springer, 2000.

[17] Eleanor Sanderson, George Davey Smith, Frank Windmeijer, and Jack Bowden. An examination of multivariable mendelian randomization in the single-sample and two-sample summary data settings. *International journal of epidemiology*, 48(3):713–727, 2019.

[18] Eleanor Sanderson, Wes Spiller, and Jack Bowden. Testing and correcting for weak and pleiotropic instruments in two-sample multivariable mendelian randomization. *Statistics in medicine*, 40(25):5434–5452, 2021.

[19] Eleanor Sanderson and Frank Windmeijer. A weak instrument f-test in linear iv models with multiple endogenous variables. *Journal of econometrics*, 190(2):212–221, 2016.

[20] John D Sargan et al. Testing for misspecification after estimating using instrumental variables. *Contributions to econometrics: John denis sargan*, 1:213–235, 1988.

[21] Jane-Ling Wang, Jeng-Min Chiou, and Hans-Georg Müller. Functional data analysis. *Annual Review of Statistics and its application*, 3:257–295, 2016.

[22] Jingshu Wang, Qingyuan Zhao, Jack Bowden, Gibran Hemani, George Davey Smith, Dylan S Small, and Nancy R Zhang. Causal inference for heritable phenotypic risk factors using heterogeneous genetic instruments. *PLoS genetics*, 17(6):e1009575, 2021.

[23] Fang Yao, Hans-Georg Müller, and Jane-Ling Wang. Functional data analysis for sparse longitudinal data. *Journal of the American statistical association*, 100(470):577–590, 2005.

[24] Qingyuan Zhao, Jingshu Wang, Gibran Hemani, Jack Bowden, and Dylan S. Small. Statistical inference in two-sample summary-data Mendelian randomization using robust adjusted profile score. *The Annals of Statistics*, 48(3):1742 – 1769, 2020.
